# Assessing *Aedes aegypti* candidate genes during viral infection and *Wolbachia*‐mediated pathogen blocking

**DOI:** 10.1111/imb.12764

**Published:** 2022-02-14

**Authors:** Leah T. Sigle, Matthew Jones, Mario Novelo, Suzanne A. Ford, Nadya Urakova, Konstantinos Lymperopoulos, Richard T. Sayre, Zhiyong Xi, Jason L. Rasgon, Elizabeth A. McGraw

**Affiliations:** ^1^ Department of Entomology and Center for Infectious Disease Dynamics The Pennsylvania State University University Park Pennsylvania USA; ^2^ Pebble Labs, Little Fly Division Los Alamos New Mexico USA; ^3^ Department of Microbiology and Molecular Genetics Michigan State University East Lansing Michigan USA; ^4^ Department of Biology and Center for Infectious Disease Dynamics The Pennsylvania State University University Park Pennsylvania USA

**Keywords:** alpha‐mannosidase, chikungunya virus, cadherin, dengue infection, *Wolbachia*‐mediated viral blocking

## Abstract

One approach to control dengue virus transmission is the symbiont *Wolbachia*, which limits viral infection in mosquitoes. Despite plans for its widespread use in *Aedes aegypti*, *Wolbachia*'s mode of action remains poorly understood. Many studies suggest that the mechanism is likely multifaceted, involving aspects of immunity, cellular stress and nutritional competition. A previous study from our group used artificial selection to identify a new mosquito candidate gene related to viral blocking; *alpha‐mannosidase‐2a* (*alpha‐Mann‐2a*) with a predicted role in protein glycosylation. Protein glycosylation pathways tend to be involved in complex host–viral interactions; however, the function of alpha‐mannosidases has not been described in mosquito–virus interactions. We examined *alpha‐Mann‐2a* expression in response to virus and *Wolbachia* infections and whether reduced gene expression, caused by RNA interference, affected viral loads. We show that dengue virus (DENV) infection affects the expression of *alpha‐Mann‐2a* in a tissue‐ and time‐dependent manner, whereas *Wolbachia* infection had no effect. In the midgut, DENV prevalence increased following knockdown of *alpha‐Mann‐2a* expression in *Wolbachia*‐free mosquitoes, suggesting that *alpha‐Mann‐2a* interferes with infection. Expression knockdown had the same effect on the togavirus chikungunya virus, indicating that *alpha‐Mann‐2a* may have broad antivirus effects in the midgut. Interestingly, we were unable to knockdown the expression in *Wolbachia*‐infected mosquitoes. We also provide evidence that *alpha‐Mann‐2a* may affect the transcriptional level of another gene predicted to be involved in viral blocking and cell adhesion; *cadherin87a*. These data support the hypothesis that glycosylation and adhesion pathways may broadly be involved in viral infection in *Ae. aegypti*.

## Graphical abstract

The expression of an *Aedes aegypti* candidate *Wolbachia*‐mediated pathogen blocking gene, *alpha‐Mann‐2a*, is affected by dengue virus (DENV) in a tissue‐ and time‐dependent manner, and *Wolbachia* infection had no effect. Following knockdown of *alpha‐Mann‐2a* expression in the midgut, DENV infection prevalence increased in *Wolbachia*‐free mosquitoes, and the same effect was observed during chikungunya virus infection. Knockdown of *alpha‐Mann‐2a* expression also decreases another candidate *Wolbachia*‐mediated pathogen blocking gene, *cadherin87a*, suggesting that both genes contribute to infection outcomes.

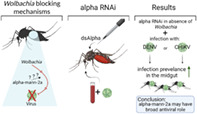

## INTRODUCTION

The mosquito *Aedes aegypti* plays an extensive role in transmitting the following viruses: dengue (DENV), Zika (ZIKV), chikungunya (CHIKV) and yellow fever, which have serious health consequences in humans globally (Gould et al., 2017). Without effective, licensed vaccines, prevention of arboviral diseases is focused on mosquito control via habitat reduction and the use of insecticides. Such interventions are difficult to implement in regions of impoverishment, urbanization and poor sanitation (Fauci & Morens, 2016). Furthermore, insecticide resistance in mosquitoes has hampered these efforts and assisted in the increase in dengue cases (Dusfour et al., 2019). It is estimated that yearly 390 million become infected with dengue, with 96 million experiencing pathological symptoms, and efforts to create a vaccine have faced difficulties due to the circulation of four different dengue serotypes across the world (Izmirly et al., 2020).

One alternative means of disease control is through the use of the bacterium *Wolbachia*, which is being released globally to decrease virus transmission to humans through population ‘suppression’ and population ‘replacement’ (Ferreira et al., 2020). *Wolbachia* are endosymbiotic bacteria that naturally infect arthropods and are maternally transmitted to offspring (Zug & Hammerstein, 2012). *Wolbachia* species native to a range of other insects have been introduced into the naturally *Wolbachia*‐free *Ae. aegypti* where they have formed stably inherited infections (McMeniman et al., 2009; Xi et al., 2005). ‘Population suppression’, involving the release of *Wolbachia*‐infected males whose offspring are not viable when mated with wild females, has led to reductions in mosquito populations where trialled in the United States and China (Crawford et al., 2020; Zheng et al., 2019). ‘Population replacement’ is another control approach and is achieved by releasing female *Ae. aegypti* that pass on *Wolbachia* to their offspring, which then spreads through the population. Female mosquitoes infected with *Wolbachia* harbour reduced dengue viral loads, exhibit longer extrinsic incubation periods or fail to transmit virus in the saliva all together (Moreira et al., 2009; Ye et al., 2015). Field trials of replacement strategies have shown some efficacy in reducing the incidence of dengue fever in humans (Carrington et al., 2018; Nazni et al., 2019; Utarini et al., 2021). *Wolbachia* also limits the replication of other arboviruses in mosquitoes, including CHIKV, ZIKV, yellow fever and West Nile (WNV), suggesting a fundamental shared mechanism that could be useful for many diseases (Joubert & O'Neill, 2017; Kambris et al., 2009; Moreira et al., 2009; van den Hurk et al., 2012).

The potential use of *Wolbachia* population replacement for biocontrol by pathogen blocking is immense. While the mechanisms of blocking are still poorly understood, there is a growing consensus that it is multifaceted (Lindsey et al., 2018). Understanding how mosquito–virus–*Wolbachia* genetic interactions dictate pathogen blocking is necessary to assure the efficacy of the biocontrol agent across a global landscape of mosquito and viral genetic diversity. In addition, understanding the mechanism will be key to developing strategies to counter the evolution of resistance against *Wolbachia* or its effects (Joubert et al., 2016), either in the mosquito or the virus. Our own work has shown that we can rapidly artificially select for differences in DENV blocking strength in mosquitoes collected from a single field release site (Ford et al., 2019), demonstrating the importance of mosquito genetic diversity in the trait's expression. Genome‐wide association (GWA) in selected lines exhibiting stronger and weaker blocking revealed a set of candidate mosquito genes that underpin blocking phenotypes. One of the top candidate genes was *alpha‐mannosidase‐2a* (*alpha‐Mann‐2a*). Alpha‐mannosidase proteins cleave mannose residues on glycoproteins as they move through the secretory pathway in the endoplasmic reticulum (ER) and Golgi, creating the substrate for the next step in a complex process of N‐linked glycosylation (Stanley, 2011). In the ER, alpha‐mannosidases also recognize mis‐folded proteins and facilitate degradation through the ER‐associated degradation pathway (Hosokawa et al., 2001). The predicted ortholog to *alpha‐Mann‐2a* in Drosophila, *alpha‐Man‐II‐b*, has also been demonstrated to have deglycosylation activity, though its role is unknown, and its increased variation compared with other *Drosophila* alpha‐mannosidases suggests a deviation in its function (Rosenbaum et al., 2014).

Alpha‐mannosidases have not directly been associated with DENV infection; however, the expression of an alpha‐mannosidase‐1 positively correlated with Hepatitis B virus during infection (Jiang et al., 2016), and a hantavirus protein colocalizes with mammalian alpha‐mannosidase‐2 protein during infection (Ravkov & Compans, 2001). Another possibility is that *alpha‐Mann‐2a* may affect the glycosylation of viral proteins rather than those of host origin. Glycoproteins are a major component of viral surface proteins and are important for CHIKV and DENV attachment to cells, and DENV secretes non‐structural glycoproteins (Banerjee & Mukhopadhyay, 2016). With no functional evidence of the role of this gene in mosquitoes, further investigation during infection is required. Since the key single nucleotide polymorphisms (SNPs) in *alpha‐Mann‐2a* identified in our GWA were located in the non‐coding regions (Ford et al., 2019), we sought to manipulate the expression of this gene to see the most effect. We therefore used RNA interference (RNAi) to assess whether reduced expression of *alpha‐Mann‐2a* had any impact on the DENV and CHIKV infection loads in mosquitoes. This gene may offer a potential target for genetic modification in the effort to create virus refractory mosquitoes.

## RESULTS

### *The effect of DENV and* Wolbachia *infection on* alpha‐Mann‐2a *expression*

**FIGURE 1:**
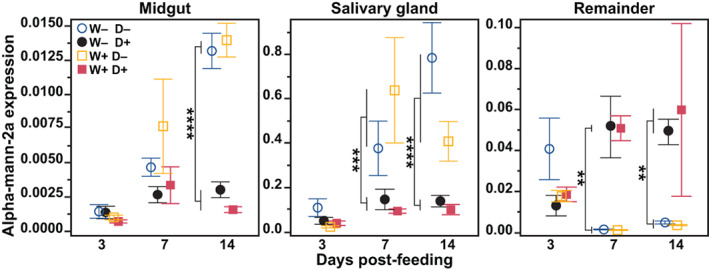
Effect of *Wolbachia* and dengue virus (DENV) infection on *alpha‐Mann‐2a* expression. At 3, 7 and 14 days post‐feeding, the midguts, salivary glands and remaining tissues (remainder) were dissected from *Wolbachia*‐free (*W*−) and *w*AlbB (*W*+) mosquitoes that fed on blood containing DENV (D+) or blood without DENV (D−). The mRNA levels of *alpha‐Mann‐2a* are expressed relative to *RpS6*. Symbols represent the mean and bars the standard error of the mean. Tukey post hoc multiple comparisons: **p < 0.01, ***p < 0.001, ****p < 0.0001. *N* = 7–8

We hypothesized that infection may regulate the expression of *alpha‐Mann‐2a* and so we measured the expression in both *Wolbachia*‐free (*W*−) and *w*AlbB mosquitoes (*W*+) that consumed either DENV‐infected (D+) or DENV‐free blood (D−). The midgut, salivary gland and remaining tissues (remainder) were collected at 3, 7 and 14 days post‐feeding for each treatment group. Expression of the candidate gene was normalized to the housekeeping gene *Rps6*. The expression levels of *alpha‐Mann‐2a* were regulated by tissue, time and DENV infection status, but *Wolbachia* had no effect (mixed effects model: tissue, time and infection *p* < 0.0001, *Wolbachia p* = 0.44, Table S1). Overall expression was lowest in the midgut and highest in the salivary glands, where on average expression levels at 7‐ and 14 days post‐feeding on blood without DENV were at least 100‐fold higher than in the other tissues (Figure 1). In the midgut, the impact of DENV was limited to 14 days post‐feeding, where expression in both wild‐type and *Wolbachia*‐infected mosquitoes decreased more than four‐fold in the presence of DENV when compared with uninfected blood fed mosquitoes (post hoc Tukey time × infection: *p* < 0.0001). Salivary gland gene expression trends were similar to that of the midgut, with DENV‐infected mosquito lines having 2.5‐ to 3‐fold lower *alpha‐Mann‐2a* expression at 7 and 14 days post‐feeding, compared with uninfected blood fed mosquitoes (post hoc Tukey time × infection: *p* = 0.001 and *p* < 0.0001, respectively). In contrast, in the remainder tissue, *alpha‐Mann‐2a* expression increased in the presence of DENV at 7 days post‐feeding and remained elevated over 12‐fold at 14 days post‐feeding for both *W*− and *W*+ mosquitoes (post hoc Tukey time × infection: *p* = 0.008 and *p* = 0.007, respectively). Overall, *Wolbachia* infection itself had no impact on *alpha‐Mann‐2a* expression and DENV impacts were tissue‐ and time‐specific. DENV infection induced differential expression of *alpha‐Mann‐2a* later in infection, decreasing expression in the midgut and salivary glands and increasing expression in the remainder tissue.

### alpha‐Mann‐2a *knockdown during DENV infection*

**FIGURE 2:**
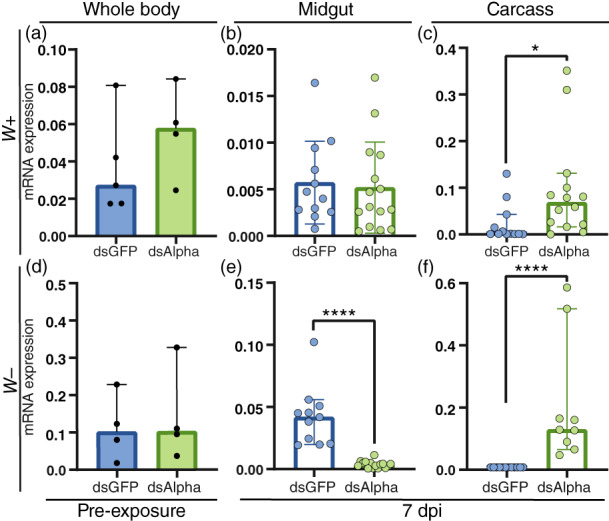
Expression of *alpha‐Mann‐2a* following RNA interference (RNAi). The expression of *alpha‐Mann‐2a* in control mosquitoes (dsGFP) and during *alpha‐Mann‐2a* RNAi (dsAlpha). (a–c) *w*AlbB mosquitoes (*W*+). (d–f) *Wolbachia*‐free mosquitoes (*W*−). (a, d) The levels of *alpha‐Mann‐2a* mRNA in whole‐body mosquitoes prior to dengue virus exposure through blood feeding (pre‐exposure) at 3 days post‐RNAi. Black circles represent individual whole‐body samples. *N* = 4–5. (b, c, e, f) Expression of *alpha‐Mann‐2a* at 7 dpi in the midgut and carcass. Blue and green circles represent individual tissues. Graphs display the relative expression to *RpS6*, bars represent the median and whiskers depict the 95% confidence intervals. Mann–Whitney: *p < 0.05, ****p < 0.0001. *N* = 9–15

We hypothesized that *alpha‐Mann‐2a* affects DENV infection and dissemination from the midgut to the body in mosquito lines with and without *Wolbachia*. To investigate the role of *alpha‐Mann‐2a* during DENV infection and *Wolbachia*‐mediated pathogen blocking, mosquitoes were injected with double‐stranded RNA (dsRNA) complimentary to *alpha‐Mann‐2a* to decrease its mRNA expression through the RNAi pathway. On day 3 following dsRNA injection, mosquitoes were fed on infectious blood and unfed mosquitoes were discarded. Midguts and the remaining carcasses containing the salivary glands were sampled at 7 days post‐infection (dpi) and again at 10 dpi, a time point when salivary glands are infected in the majority of mosquitoes (Salazar et al., 2007). The *Wolbachia*‐mediated blocking significantly reduced DENV‐infected individuals as expected (Figure S1); however, knockdown was not effective in any of the tissues of *Wolbachia*‐infected mosquitoes (Figure 2a–c). We also checked the *Wolbachia* densities across treatment groups and found that RNAi had no effect on *w*AlbB densities (Figure S2). We did see effective knockdown in the midgut of wild‐type mosquitoes infected with DENV 7 dpi, with 90% of the control levels of expression suppressed (Figure 2e). In the carcass the transcript levels surprisingly increased compared to controls (Figure 2f).

**FIGURE 3:**
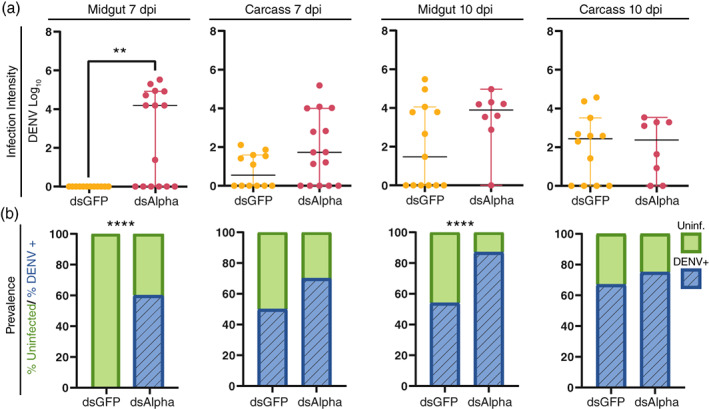
Dengue virus (DENV) infection during RNA interference knockdown of *alpha‐Mann‐2a*. DENV intensity and prevalence were detected by absolute qRT‐PCR in midgut and carcass samples at 7 and 10 dpi. (a) Infection intensity (genome copy number) over time. Lines mark the median, circles represent DENV quantities in individual dsGFP (yellow) or dsAlpha (magenta) tissue samples, and whiskers depict the 95% confidence intervals. Mann–Whitney test, **p < 0.01. (b) Prevalence (presence or absence) of DENV. Bars contain the percentage of mosquitoes uninfected (green) and DENV infected (blue). Binary logistical regression for Midgut: *p* < 0.0001, likelihood ratio test for treatment, ****p < 0.0001. Binary logistical regression for carcass: *p* = 0.48. *N* = 8–15

Knockdown of *alpha‐Mann‐2a* significantly increased the intensity (viral load) of DENV infection in the midgut at 7 dpi (Figure 3a, Mann–Whitney test *p* < 0.01). Of those mosquitoes, the DENV prevalence (percent infected) was 60% higher in *alpha‐Mann‐2a* knockdown midguts, and prevalence also increased by 33% at 10 dpi in the midgut (Figure 3b, likelihood ratio test, treatment *p* < 0.0001). Overall, *alpha‐Mann‐2a* knockdown was associated with increased DENV prevalence, suggesting that *alpha‐Mann‐2a* interferes with DENV infection in the midgut. When DENV prevalence increased at 7 dpi in the midgut, transcript levels of *alpha‐Mann‐2a* were reduced by more than 90%, confirming that silencing by RNAi was occurring (Figure 2e, Mann–Whitney *p* < 0.0001). The lack of the effect in the carcass for either intensity (Figure 3a) or prevalence (Figure 3b) is not surprising given a lack of gene expression knockdown (Figure 2f). Additionally, this could indicate differential roles of this enzyme in the midgut and carcass, which would be supported by our expression data (Figure 1). Overall, a single dose of dsRNA targeting *alpha‐Mann‐2a* (dsAlpha) prior to infection can increase DENV prevalence in the midgut at 7 and 10 dpi. This is important for potential downstream effects on DENV dissemination and invasion of the salivary glands.

### alpha‐Mann‐2a *knockdown during CHIKV infection*

Next, we were interested in comparing the potential functional role of *alpha‐Mann‐2a* in the presence of an alphavirus vs. a flavivirus. We used the same dsRNA for knockdown as above and infected mosquitoes with CHIKV. Knowing that CHIKV infection proceeds faster, we took time points at what would be biologically comparable in terms of viral dissemination.

**FIGURE 4:**
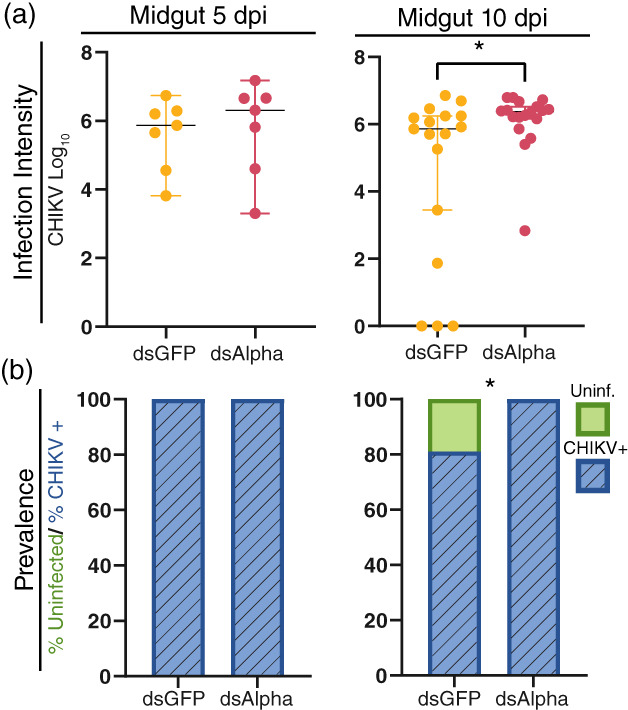
Chikungunya virus (CHIKV) infection during RNA interference knockdown of *alpha‐Mann‐2a* in the midgut. CHIKV intensity and prevalence were detected by absolute qRT‐PCR in midgut samples at 5‐ and 10 dpi. (a) Infection intensity (genome copy number) over time. Lines mark the median, circles represent CHIKV quantities in individual dsGFP (yellow) or dsAlpha (magenta) tissue samples, and whiskers depict the 95% confidence intervals. Mann–Whitney test, *p < 0.05. (b) Prevalence (presence or absence) of CHIKV. Bars contain the percentage of mosquitoes uninfected (green) and CHIKV infected (blue). Binary logistical regression for Midgut: *p* = 0.03, likelihood ratio test for treatment, *p = 0.03. At 5 dpi *N* = 7 and at 10 dpi *N* = 17–18

In wild‐type mosquitoes at 10 dpi, the intensity of CHIKV infection was significantly higher in the *alpha‐Mann‐2a* knockdown mosquito midguts (Figure 4a). This was largely driven by the increase in the number of infected mosquitoes in this group (Figure 4b), and similar to the effects observed during DENV infection (Figure 3). By 10 dpi in control midguts, the prevalence of CHIKV was 81%, and *alpha‐Mann‐2a* knockdown increased CHIKV prevalence to 100% (likelihood ratio test, *p* = 0.03). Though similar to the effects observed during DENV infection, comparatively the trend was not as strong. The overall prevalence and intensity of CHIKV infection were higher than in DENV control mosquitoes and likely due to the higher titre that was harvested for the bloodmeal. The expression of *alpha‐Mann‐2a* in dsAlpha mosquitoes was reduced by 75% prior to infection; however, by 5 dpi expression was not significantly lower (Figure S3). This recovery of expression could be another reason the effects are not as strong. As with DENV infection, no effects were observed in the carcass (Figure S4). Overall, the data supports that *alpha‐Mann‐2a* limits viral prevalence in the midgut during both DENV and CHIKV infections.

### alpha‐Mann‐2a *and* cadherin*87a interactions*

Since the conventional role of alpha‐mannosidase‐2 is to help convert a high mannose glycosylated structure to a complex mannose structure, we hypothesized that the role of *alpha‐Mann‐2a* may function through its enzymatic activity on another protein. For multiple reasons we looked into *cadherin87a* (AAEL023845), the next top candidate gene from our previous study (Ford et al., 2019). In addition to both genes containing genetic variation that associated with the strength of DENV blocking, some cadherins require glycans in order to mediate cell adhesion (Lommel et al., 2013). Therefore, it is plausible there could be functional interactions with *alpha‐Mann‐2a*. Second, there are data from mammalian systems showing that decreasing the expression of an alpha‐mannosidase gene also changed the expression of an E‐cadherin (Tian et al., 2008). Finally, these genes are close in proximity to each other, just under 1.5 Mb apart. Cadherins are a large and diverse family of proteins that all have extracellular cadherin repeat domains and many roles including cell–cell adhesion, cell polarity and intracellular signalling (Halbleib & Nelson, 2006). Classical cadherins form adherens junctions with nectin proteins, and nectin is bound by poliovirus, human herpes simplex virus and the measles virus in order to cross the epithelium (Mateo et al., 2015). Though a cadherin protein is yet to be identified as a viral receptor in any organism, multiple mosquito cadherins have been identified that bind DENV proteins (Colpitts et al., 2011; Muñoz et al., 2013). We were interested to see if knockdown of *alpha‐Mann‐2a* affected expression of *cadherin87a*.

**FIGURE 5:**
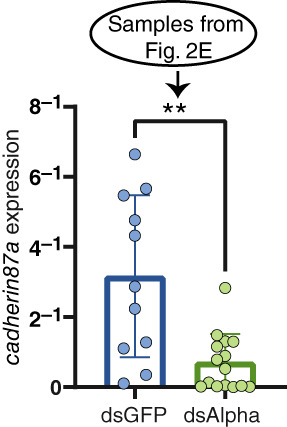
Expression of *cadherin87a* during *alpha‐mann‐2a* RNA interference. Transcript levels of *cadherin87a* following dengue virus infection at 7 dpi in the midgut of control (dsGFP) and dsAlpha injected mosquitoes. Graphs display the relative expression compared with *RpS6*. Bars represent the median and whiskers depict the 95% confidence intervals. Circles represent individual midguts. Mann–Whitney: **p = 0.003, *N* = 11–15. The graph of *alpha‐mann‐2a* transcription in these same samples is found in Figure 2e

We checked the levels of *cadherin87a* in our dsAlpha treated mosquitoes. When *alpha‐Mann‐2a* expression decreased by more than 90% at 7 dpi in the midguts of dsAlpha mosquitoes (Figure 2e), *cadherin87a* expression also decreased by 77% (Figure 5). These results suggest that the increases in infection upon *alpha‐Mann‐2a* knockdown may result from a decrease of both *alpha‐Mann‐2a* and *cadherin87a* in the midgut. These results where surprising since we suspected these genes to have opposing functions during infection. The decrease in *cadherin87a* expression upon *alpha‐Mann‐2a* KD suggests that there could be direct or indirect interactions in the functions of these genes during infection. For example, the functional activity of *alpha‐Mann‐2a* may affect *cadherin87a* protein turnover, and therefore expression, or decreases in *cadherin87a* expression may be indirect effects from increasing DENV during *alpha‐Mann‐2a* knockdown.

**FIGURE 6:**
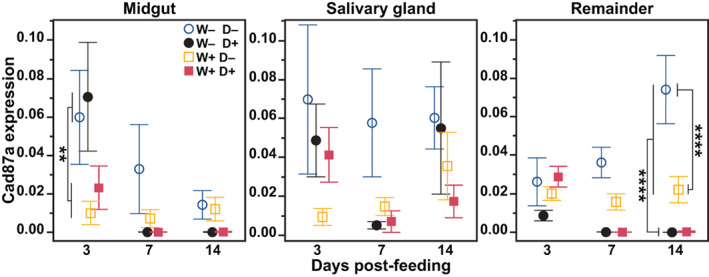
Effect of *Wolbachia* and dengue virus (DENV) infection on *cadherin87a* expression. At 3, 7, and 14 days post‐feeding, the midguts, salivary glands and remaining tissues (remainder) were dissected from *Wolbachia*‐free (*W*−) and *w*AlbB (*W*+) mosquitoes that fed on blood containing DENV (D+) or blood without DENV (D−). The mRNA levels of *cadherin87a* are expressed relative to *RpS6*. Symbols represent the mean and bars the standard error of the mean. Tukey post hoc multiple comparisons: **p < 0.01, ****p < 0.0001. *N* = 7–8

We then went back to our initial expression experiment and checked cadherin expression in these same tissues. *Cadherin*87a expression was impacted by tissue, time, infection and *Wolbachia* (mixed effects model *Wolbachia*, time, and infection: *p* < 0.0001, tissue *p* = 0.02). Overall expression levels did not vary highly across tissues (Figure 6). In the midgut DENV infection had no impact on cadherin expression (mixed effects model time × infection × *Wolbachia*: DENV *p* = 0.37), and *Wolbachia* decreased expression at 3 days post‐bloodmeal by 2.5‐fold (post hoc Tukey: time × *Wolbachia p* < 0.009). In the salivary glands of DENV‐free mosquitoes, *Wolbachia* also reduced expression post‐bloodmeal across all time points (post hoc Tukey: *Wolbachia p* = 0.004). At 14 dpi in the remainder tissue, *Wolbachia* reduced *cadherin*87a by 3‐fold, and DENV also reduced expression in both *W*− and *W*+ mosquitoes by 60‐fold or more (post hoc Tukey infection × *Wolbachia*: *Wolbachia* and infection *p* < 0.0001). In summary, the presence of *Wolbachia* reduced *cadherin*87a expression in the midgut and DENV reduced expression in the remainder. In the midgut at 14 dpi, *cadherin87a* was lower in the DENV‐infected samples and trending in the same direction that *alpha‐Mann‐2a* does in response to DENV in the midgut, though not significantly different. There are also significant differential expression patterns between *alpha‐Mann‐2a* and *cadherin87a*. In the remainder, at 14 dpi, DENV had opposite effects, increasing *alpha‐Mann‐2a* and decreasing *cadherin*87a expression (Figures 1 and 6).

## DISCUSSION

Current control approaches releasing *Wolbachia*‐mosquitoes aim to decrease mosquito susceptibility to viral infection or to reduce the number of wild mosquitoes (Ferreira et al., 2020). Both strategies require large‐scale mosquito rearing facilities for establishment and long‐term monitoring for success (Ritchie & Staunton, 2019). These high investment approaches are proving to have high returns with respect to effectively crashing mosquito populations and for reducing the incidence of dengue fever in humans via pathogen blocking (Carrington et al., 2018; Nazni et al., 2019; Zheng et al., 2019). Insights into how pathogen blocking operates at the molecular level may provide strategies and solutions for maintaining the success of *Wolbachia* in the face of evolutionary or ecological hinderances. Additionally, independently targeting the mosquito pathways acted upon by *Wolbachia* could lead to GM technology that is more accessible and easier to deploy in mosquitoes than symbiont‐based methods.

A range of mechanisms has been proposed to explain *Wolbachia*‐mediated virus blocking. The growing consensus is that the effect is likely multifaceted (Lindsey et al., 2018). There is some evidence that mosquito immunity (Pan et al., 2012; Terradas et al., 2017), cellular stress (Wong et al., 2015) and competition for space or nutritional resources (Caragata et al., 2013, 2016) as well as other factors could play a role. We recently sought to identify the basis of viral blocking using an unbiased approach by artificially selecting upon the natural variation observed in this phenotype (Ford et al., 2019). By identifying SNPs that differentiated low‐ and high‐blocking selected lines, we identified a suite of new candidate loci associated with these phenotypes. Few of the genes we identified containing key SNPs recapitulated previously proposed hypotheses, although there was some evidence of selection driving variation in oxidative stress genes (Ford et al., 2019). The two top candidate genes, surprisingly, pointed to potential impacts on protein glycosylation and or cell adhesion.

In this study, we sought to obtain functional data on the role of *alpha‐Mann‐2a*, in virus replication in the mosquito by using RNAi. We took a practical and conservative approach in our design of gene knockdown, by using a single dose of dsRNA prior to infection and assessing infection at later time points when the virus has disseminated. A caveat to this design is that we could have missed earlier effects on infection or any larger differences that may have waned over time. We chose this approach first, since the gene being studied was identified through experimental infection via the body cavity, therefore initially bypassing the midgut barrier, and second to see if a single dose of dsRNA could induce RNAi and have lasting effects throughout an infection. With oral delivery systems being developed for larval and adult stage mosquitoes (Wiltshire & Duman‐Scheel, 2020), RNAi‐based strategies have the potential to be deployed for mosquito control if their impacts can last beyond several days. Furthermore, different methods of RNAi induction and delivery, such as microbial‐based delivery, could be pursued to enhance the efficiency of knockdown. Our goal was to initially assess if *alpha‐Mann‐2a* is a good target for adult stage manipulation of infection.

Although we have no evidence of what *alpha‐Mann‐2a* and *cadherin87a* are doing in the mosquito, there is information in the broader literature within these gene families about localization, activity and function, which can inform on how they may affect infection. Both, *alpha‐Mann‐2a* and *cadherin87a*, are members of large gene families that are further divided into subfamilies based on sequence domains, localization and protein activity (Gonzalez & Jordan, 2000; Oda & Takeichi, 2011). Individual genes in these families may have novel, distinct functions or provide redundancies in functionality with other genes. In mosquitoes, alpha‐mannosidases have not been studied, and a search for ‘alpha‐mannosidase’ in the *Ae. aegypti* genome (AaegL5.0) results in 11 alpha‐mannosidase genes, including some that are specifically predicted to be lysosomal or ER degradation enhancing. The exact role of the *alpha‐Mann‐2a* ortholog in *Drosophila melanogaster* is unknown, but it is required for proper maturation of a rhodopsin protein in the fly eye (Rosenbaum et al., 2014). A search for ‘cadherin’ in the *Ae. aegypti* genome results in more than 20 genes and *cadherin87*a is 1 of 7 that are categorized as protocadherins or neural cadherins. Another cadherin gene in *Ae. aegypti* has been identified as a receptor for toxins released by the bacterium *Bacillus thuringiensis*. It is expressed in the digestive tract and essential for development. The strongest localization of this protein was on the apical side of the epithelia and varied in tissue location across developmental stages, with adult females showing highest localization in the foregut (Chen et al., 2020). This gene's distinct expression dynamics and localization underline the diverse function of genes in this family. Further work into the spatial and temporal dynamics of *alpha‐Mann‐2a* and *cadherin87a* will inform on their roles during infection. In particular, the DENV‐induced increase in *alpha‐Mann‐2a* in the tissues of the body, which are opposite of the decreases in expression observed in the midgut and salivary glands, could be reflective of tissue differences or differences in pathogen interactions. Furthermore, these differences may have contributed to the inability to capture an observed knockdown in the carcass. The carcass also includes hemocytes, fat body, the heart, aorta, muscles and additional tissues in which *alpha‐Mann‐2a* may be expressed, and the time course of knockdown could proceed at a different rate.

In keeping with our data on *alpha‐Mann‐2a*, we propose two potential modes of action for its involvement in viral infection. The first hypothesis is that alpha‐Mann‐2a enzymatic activity acts upon viral proteins. The second is that alpha‐Mann‐2a enzymatic activity acts upon mosquito proteins that alter infection. In support of the first hypothesis, the presence of N‐linked glycosylation on flavivirus proteins directly impacts binding to factors on the cell's surface (Carbaugh & Lazear, 2020). N‐linked glycosylation can be further classified as high mannose, which is the first step in the stepwise process to further becoming classified as complex mannose (Yap et al., 2017). Alpha‐mannosidase‐2 is required in the first step to convert a high mannose glycosylated structure to a complex mannose structure. Insects and mammals have different glycosylation pathways, and therefore glycosylation patterns are different in these two hosts (Hacker et al., 2009). Analyses of N‐linked glycans on DENV detect high mannose glycans as a majority component when propagated in mosquito cells, and DENV derived from mammalian cells have more complex glycans (Hacker et al., 2009; Lei et al., 2015). This would suggest an absence of alpha‐mannosidase activity on DENV propagated in insect cells. Potentially the addition of complex N‐linked glycans facilitated by alpha‐Mann‐2a could lead to decreased infectivity through changes in protein binding, folding, localization or secretion. The presence of N‐linked glycosylation at a specific position on the WNV Envelope protein reduced viral infectivity in *Aedes albopictus* C6/36 mosquito cell lines, and this effect was stronger in mosquito cells compared with mammalian (Hanna et al., 2005). In human cells, WNV interactions with the C‐type lectin viral attachment factor DC‐SIGN have been shown to be dependent on the presence of high mannose residues on envelope proteins (Carbaugh & Lazear, 2020). Though this receptor is not present in insects, other C‐type lectins promote DENV, Japanese encephalitis virus and WNV infection (Caragata et al., 2019). The studies mentioned above have compared non‐glycosylated DENV to glycosylated or comparatively studied mosquito versus mammalian derived virus; however, it is unknown if altering a high mannose glycan to a complex mannose, as would be facilitated by alpha‐mannosidase, would change infectivity in mosquitoes. Lastly, it is also possible that high mannose glycosylation of viral proteins results in an evasion of immune responses as has been shown for both flaviviruses and alphaviruses in mammalian cells (Rogers & Heise, 2009), and therefore alpha‐Mann‐2a activity could result in increasing virus vulnerability to immune responses.

Our second hypothesis is that alpha‐Mann‐2a activity may alter the glycosylation of mosquito proteins. This could occur through altering the glycosylation of proteins involved in the viral response or that facilitate infection. Furthermore, the enzymatic activity of alpha‐Mann‐2a could lead to protein turnover, as inhibitors of alpha‐mannosidase lead to decreased degradation of glycoproteins (Segal & Winkler, 1984). In summary, alpha‐Mann‐2a may destabilize the activity or recycling of a protein that facilitates viral infection.

The enzymatic activity of alpha‐Mann‐2a may even act on cadherin87a, as the expression of *cadherin87a* also decreased during knockdown of *alpha‐Mann‐2a*. It is not clear how decreasing *alpha‐Mann‐2a* would also decrease *cadherin87a* expression, and this would be unexpected based on our hypotheses of opposing function. However, our expression data show opposite directional expression changes in the remainder in the presence of DENV, however this does not occur in the midgut tissue (Figures 1 and 6).One possibility of the observed effects in our KD is that the alpha‐Mann‐2a protein may destabilize the activity or recycling of the cadherin protein. This puts forth the hypothesis that when cadherin87a is in a high mannose glycosylation state (in the absence of *alpha‐Mann‐2a*) it is more stabilized or recycled, and there is a decreased need to express more *cadherin87a*, meaning that the *cadherin87a* expression data may not be reflective of protein levels or activity. In support, classical cadherin activity requires high mannose glycosylation for proper cadherin‐based adhesion in cell junctions (Lommel et al., 2013; Vester‐Christensen et al., 2013). Last, cadherins are trafficked, sorted and recycled to the plasma membrane through endocytosis (Brüser & Bogdan, 2017), and glycosylation could play a role in these processes. Another possibility is that indirectly the effects of *alpha‐Mann‐2a* KD on DENV then affect *cadherin87a* expression.

We showed that *w*AlbB decreased expression of *cadherin87a* in the midgut, supporting that lower expression could decrease infection; however, DENV also decreased expression in the tissues of the body at later time point in infection. Altogether, *cadherin87a* may have different roles across tissues and time, and a closer look at protein localization is needed to further assess potential interactions. Interestingly, the *cadherin87a* gene is 869,996 bp with 27 exons and 5 predicted transcripts (AaegL5.0), so there could also be transcript specificity. Previous studies have identified multiple mosquito cadherins that bind DENV proteins (Colpitts et al., 2011; Muñoz et al., 2013), and one cadherin (AAEL001196) facilitated DENV infection in the *Ae. aegypti* Aag‐2 cell line. However, *w*AlbB actually increased the expression of this cadherin gene, and knockdown did not affect pathogen blocking of DENV (Lu et al., 2020). In human cells, an E‐cadherin indirectly facilitates the entry of Hepatitis C virus as it is required for proper localization of receptor proteins in the nearby tight junctions on the cell surface (Li et al., 2016). Interestingly, later in infection, the expression of this E‐cadherin decreased and correlated with increased markers for disease progression in infected cells, likely due to decreased epithelial integrity. This suggests that a single cadherin could have a multifaceted role during an infection, initially facilitating cell entry and later being targeted to disrupt cell to cell adhesions to increase permeability for dissemination. Changes in mosquito cell integrity during infection have not been described; however, in humans haemorrhagic fever during DENV infection associates with decreases in vascular cadherins involved in membrane permeability (Dewi et al., 2008; Kanlaya et al., 2009).

Interestingly, *alpha‐Mann‐2a* gene knockdown by RNAi only occurred in the absence of *Wolbachia*. This is intriguing since we hypothesize that *alpha‐Mann‐2a* decreases viral infection. We show that DENV changes *alpha‐Mann‐2a* expression but the presence of *Wolbachia* had little effect. This is not completely surprising since the variation that correlates with pathogen blocking in this gene occurs within introns (Ford et al., 2019). This suggests that *Wolbachia* may affect splicing and not expression. Recent work in *D. melanogaster* identified alternative splicing of genes in the presence of *Wolbachia* and Sindbis virus (Bhattacharya et al., 2017). So further assessment of the role of these genes in pathogen blocking will be done in combination with transcriptomics looking at splicing variation. *Wolbachia* could also potentially alter the rate of protein turnover, which would indirectly affect transcription and the ability to knockdown a transcript. This is supported by the possibility of overlap in physical space, as alpha‐mannosidase proteins associate with the ER and Golgi, and *Wolbachia* physically interacts with the ER and alters its subcellular organization (Fattouh et al., 2019). Furthermore, these potential interactions could facilitate the pathogen blocking phenotype by *Wolbachia* through increasing or stabilizing alpha‐Mann‐2a activity.

Both *alpha‐Mann‐2a* and *cadherin87a* remain as interesting candidates in *Aedes*, *Wolbachia* and arbovirus tripartite interactions. The gene *alpha‐Mann‐2a* impacts viral infection and has the potential to regulate or participate in cellular processes that could be crucial for both *Wolbachia* and arboviruses. Further analysis on the cellular level in combination with enzyme inhibitors and protein markers will distinguish if these genes have roles atypical of other proteins in these super families. Finally, our data indicate that adhesion and glycosylation pathways may broadly be involved in viral infection in *Ae. aegypti*.

## EXPERIMENTAL PROCEDURES

### Mosquito rearing

*Ae. aegypti* mosquitoes previously infected with the *w*AlbB strain of *Wolbachia* were backcrossed for seven generations into a wild line, AFM, isolated from Merida Mexico in 2017. All mosquitoes were reared and maintained at 27°C, 60% relative humidity and a 12 h:12 h light/dark photoperiod. Larvae were reared in deionized water and fed fish food (TetraMin) ad libitum. Prior to eclosion, the pupal stage was transferred in small cups of water to a cage, and emerging adults were fed 10% sucrose.

### Virus cultivation and mosquito blood feeding

The dengue virus serotype 2 (DENV‐2) strain JAM 1409 (Bennett et al., 2002) was propagated in *A. albopictus* C6/36 cells, as previously described (Terradas et al., 2017). Briefly, C6/36 cells were maintained in RPMI medium 1640 (Life Technologies) supplemented with 10% heat‐inactivated fetal bovine serum (FBS), (Life Technologies), 20 mM HEPES buffer (Sigma‐Aldrich) and 1% Penicillin–Streptomycin (Life Technologies). On the day of infection, FBS was reduced to 2% in the medium and a frozen aliquot of DENV‐2 was introduced at a multiplicity of infection (MOI) of 0.002 virus particles per cell to a T75 flask at 80% confluency. Flasks were incubated at 27°C for 7 days, and the supernatant was harvested at a titre of 1.0 × 10^5^ focus forming units per ml (FFU protocol below) and mixed with human blood in a ratio of 1:1. The CHIKV strain 20,235‐St. Martin 2013 (BEI Resources) was inoculated into flasks of C6/36 cells at 80% confluency at an MOI of 0.067 virus particles per cell and followed the protocol described for DENV infection. At 2 dpi, the supernatant was harvested at a titre of 8.7 × 10^5^ FFU/ml and mixed 1:1 with human blood. Female mosquitoes of both lines were aged to 7 days and deprived of sugar for 24 h prior to being offered blood. Mosquitoes were fed the virus laden blood meal of either DENV or CHIKV using glass bell feeders covered with a sausage casing, warmed to 37°C with water from a water bath. After 2 h, the mosquitoes were anaesthetized on ice and unfed or partially fed mosquitoes were removed. All CHIKV work was carried out in the Pennsylvania State Pell BSL‐3 Laboratory.

### Dissections

Prior to dissection, mosquitoes were anaesthetized with triethylamine (Sigma‐Aldrich) via exposure to a moistened 47 mm circular filter paper. The head was removed with tweezers, followed by removal of the salivary glands from the thorax, and the midgut from the abdomen using insect pins in phosphate‐buffered saline (PBS). The remaining thorax and abdominal tissues are referred to as the remainder, and in cases where the salivary glands were left intact, we refer to as the carcass.

### RNA and DNA extractions

Individual tissues from single mosquitoes were placed in 300 μl of TriReagent (Sigma‐Aldrich), or 200 μl for salivary glands, and homogenized on a Bead Ruptor Elite (Omni International, USA) using a 2.8 mm ceramic bead. Total RNA was extracted with the Direct‐zol RNA 96 Magbead Zymo kit (Zymo Research) according to the manufacture's protocol on a MagMAX Express 96 system (Applied biosystems), and RNA was eluted in 50 μl RNase free water. RNA was treated with 5 units of DNase I (Sigma‐aldrich) at room temperature (RT) for 15 min, followed by inactivation with 50 mM EDTA at 70°C for 10 min. To measure *Wolbachia* DNA, RNA and DNA combination extractions were performed using the column‐based Direct‐zol DNA/RNA Miniprep kit. RNA was eluted in 50 μl RNase free water, followed by DNA elution in 50 μl of Direct‐zol DNA Elution Buffer, and RNA was treated with DNase I (as above).

### *Gene and* Wolbachia *quantification*

All quantitative polymerase chain reactions (qPCR) were carried out on a LightCycler 480 Real‐Time machine (Roche). For gene quantification, mRNA was used in a one‐step reverse transcriptase‐qPCR (qRT‐PCR) reaction. Using qScript One‐step SYBR Green qRT‐PCR (Quantabio) according to the manufacturer's protocol, the synthesis of cDNA occurred at 50°C for 5 min followed by Taq inactivation at 95°C for 2 min. Amplification and quantification cycled 45 times at 95°C for 3 s and 60°C for 30 s, and the final cycle was followed by a melting curve analysis. Gene‐specific primers were used at a final concentration of 0.2 μM for amplification of target genes (Table S2), and the insect gene *40S Ribosomal protein S6* (*Rps6*) was used as a housekeeping gene (Pan et al., 2012). For *w*AlBb quantification by qPCR, DNA samples were amplified using PerfeCTa SYBR Green SuperMix (Quantabio) according to the manufacturer's protocol and genomic primers specific to *w*AlBb and the mosquito *40S Ribosomal protein S17*, and thermocycling conditions as previously published (Dutra et al., 2021). Gene expression and *Wolbachia* levels were calculated by the 2^−ΔCt^ method (Livak & Schmittgen, 2001).

### Virus quantification

DENV was quantified using TaqMan Fast Virus 1‐step Master Mix (Thermo Fisher Scientific) for qRT‐PCR in 10 μl reaction volumes with DENV specific primers and probes (Table S2) (Ye et al., 2014). The following protocol was used: reverse transcription at 50°C for 5 min, followed by 95°C for 20 s, and amplification cycling at 95°C for 3 s and 60°C for 30 s. A standard reference curve of known quantities of a DENV‐2 genomic fragment was used for absolute qRT‐PCR. The DENV‐2 genomic fragment was inserted into a plasmid and transformed into *Escherichia coli* as previously described (Ye et al., 2014). The linearized and purified fragment was serially diluted ranging from 10^7^, 10^6^, 10^5^, 10^4^, 10^3^, 10^2^, and 10 copies and were used to create a standard curve of DENV amplification. The standard curve was run in duplicate on each 96‐well plate, and the limits of detection were set at 10^2^ copies. CHIKV was quantified using the same reagents and protocols as DENV above, with CHIKV specific primers and probes (Table S2) (Rückert et al., 2017). A 140 bp fragment was amplified and cloned into the PGEM‐T vector system (Promega, Madison, WI), and the plasmid was transformed into *E. coli*. Plasmid was extracted by phenol‐chloroform, linearized by restriction enzyme digest with *Pst*I (NEB, Ipswich, MA) and a standard curve of 10^8^, 10^7^, 10^6^, 10^5^, 10^4^, 10^3^, 10^2^, 10, 5, 1 was serial diluted based on the copy number obtained using a Qubit fluorometer (Thermo Fisher Scientific). The limits of detection of CHIKV were set at 10 copies.

### Focus forming unit assay

Viral titres were quantified by FFU in Vero cells (Brustolin et al., 2018; Payne et al., 2006). Cells were plated and grown for 24 h to confluency in 96‐well plates at 37°C with 5% CO_2_. The next day, cells were prepared by washing in RPMI medium (described above) without FBS. Viral supernatant or homogenized mosquito bodies were serially diluted 10‐fold in medium at concentrations from 10^−1^ to 10^−6^ and added to cells in 30 μl volumes. Plates were incubated for 2 h at 27°C. After incubation, cells were washed with medium and overlaid with 100 μl of 1% methylcellulose in RPMI with 5% FBS and further incubated for 24 h (CHIKV) or 48 h (DENV). Finally, cells were fixed in 4% paraformaldehyde for 10 min, washed in PBS, blocked and permeabilized in 2% bovine serum albumin with 0.1% triton‐X for 30 min at RT, and washed again with PBS. Virus was labelled for 2 h at RT with monoclonal anti‐DENV type 2 envelope protein Clone 3H5‐1 (BEI Resources, Manassas, VA), followed by three washes in PBS. The secondary antibody, Alexa‐488 goat anti‐mouse IgG (Invitrogen, Life Science), was added at a dilution of 1:500, and plates were incubated in the dark for 1 h. Fluorescent foci were visualized and counted on an Olympus BX41 microscope equipped with an UPlanFI ×4 objective and a FITC filter and were used to calculate FFU/ml.

### RNA‐interference gene silencing

The *Ae. aegypti* gene *alpha‐Mannosidase‐2a* (AAEL004389) was targeted and silenced by RNAi. We designed many primer sets to target different regions of *alpha‐Mann‐2a* within exons 4–9. Knowing that this gene likely has a high amount of variation, and experiencing multiple primer sets that did not amplify cDNA, we designed the primers in areas where there were low numbers of SNPs based on genomic sequencing (Ford et al., 2019). Additionally appending the T7 promoter sequence for dsRNA synthesis requires a specific nucleotide sequence. To achieve the best binding, we aligned *alpha‐Mann‐2a* with the predicted ortholog in *Anopheles gambiae* (AGAP0004032) and chose conserved GGG sequences between these two species for the forward primer T7 binding location and then used the same approach to design the reverse primers. We also designed our dsRNAs to be over 400 bp in length and therefore would be processed into many siRNAs. Gene‐specific dsRNA was synthesized using the MEGAscript T7 transcription kit (Invitrogen) as previously described (Sigle & Hillyer, 2018). Briefly, templates were amplified from mosquito cDNA using PCR and primers appended with the T7 promoter sequence (Table S2). PCR products were gel purified using the PureLink Quick Gel Extraction kit (Invitrogen). Approximately 1 μg of template was used to synthesize dsRNA following the manufacturer's protocol. The dsRNA was purified by phenol/chloroform precipitation and resuspended in sterile PBS. Adult mosquitoes at 4 days post‐eclosion were anaesthetized on ice and 69 nl of dsRNA (300 ng final concentration) in PBS was injected into the hemocoel through the thoracic anepisternal cleft. Only two out of five designed dsRNAs made it through our stringent requirements and both targeted exon 9. We proceeded with the dsAlpha used in this study based on preliminary KD efficiencies being the greatest. As a control, dsGFP was injected in the same cohort of mosquitoes (Pan et al., 2012). At 3 days post‐RNAi, mosquitoes were offered an infectious bloodmeal as described above. Following infection, the midgut was separated from the carcass and each tissue was individually placed in TriReagent as above and stored at −80°C until extracted.

### Statistical analysis

For gene expression experiments with 4‐factors, a mixed effects model was run (Wolb +/−, DENV +/−, tissue and time), followed by a tissue‐specific 3‐factor model (Wolb +/−, DENV +/−, and time) with post hoc Tukey comparisons of the identified significant factors. Data were tested for normality using the Kolmogorov–Smirnov test. All significant differences in gene expression are two‐fold or greater changes in expression. Infection prevalence was analysed for each tissue using a binary logistical regression model with the presence or absence of infection as the response and with the variables of treatment (RNAi: dsGFP vs. dsAlpha) and time (dpi) as predicting effects. The significance of the effects of time and treatment on prevalence were compared using a likelihood ratio test. The Mann–Whitney test was used for *t*‐test comparisons of non‐parametric data. Analyses were performed using prism 6 Software (GraphPad, La Jolla, CA), with the exception that General linear models and logistical regression were performed in jmp pro 15 (SAS, Cary, NC). Effects were deemed significantly different at *p* < 0.05.

## CONFLICT OF INTEREST

The authors declare no potential conflict of interest.

## AUTHOR CONTRIBUTIONS

**Leah T. Sigle**: Conceptualization, formal analysis, investigation, methodology, project administration, validation, visualization, writing‐original draft, writing‐review and editing. **Matthew Jones**: Investigation, methodology, validation, writing—review and editing. **Mario Novelo**: Investigation, validation, writing—review and editing. **Suzanne A. Ford**: Investigation, writing—review and editing. **Nadya Urakova**: Methodology, resources, writing—review and editing. **Konstantinos Lymperopoulos**: Conceptualization, funding aquisition. **Richard T. Sayre**: Conceptualization, funding aquisition. **Zhiyong Xi**: Conceptualization, formal analysis, funding aquisition, methodology, project administration, resources, supervision, validation, writing—review and editing. **Jason L. Rasgon**: Conceptualization, formal analysis, funding aquisition, methodology, project administration, resources, supervision, validation, writing—review and editing. **Elizabeth A. McGraw**: Conceptualization, formal analysis, funding aquisition, methodology, project administration, resources, supervision, validation, visualization, writing—original draft, writing—review and editing.

## Supporting information


**Figure S1.** *Wolbachia* mediated DENV‐blocking during *alpha‐Mann‐2a* RNAi. DENV intensity and prevalence were detected by absolute qRT‐PCR in midgut and carcass samples at 10 and 14 dpi. A. Infection intensity (genome copy number) overtime. Lines mark the median, circles represent DENV quantities in individual dsGFP (yellow) or dsAlpha (magenta) tissue samples, and whiskers depict the 95% confidence intervals. Mann‐Whitney test, not significant. B. Prevalence (presence or absence) of DENV. Bars contain the percentage of mosquitoes uninfected (green) and DENV infected (blue). Binary logistical regression for Midgut: *P* = 0.09. Binary logistical regression for Carcass: *P* = 0.07. N = 20‐25.
**Figure S2**. *Wolbachia* levels 3 days post‐RNAi. The levels of *w*AlbB in whole body mosquitoes was similar across treatment groups at 3 days post‐dsRNA injection and prior to DENV infection. Mann‐Whitney test, dsAlpha *P* = 0.7. Bars represent the median and whiskers depict the 95% confidence intervals. N = 3.
**Figure S3**. Expression of *alpha‐Mann‐2a* following RNAi and infection with CHIKV. A. Levels of *alpha‐Mann‐2a* expression in mosquitoes at 3 days post‐RNAi (pre‐exposure) compared to control mosquitoes (dsGFP injected). Circles represent individual whole‐body samples. N = 4‐5. B‐C. Expression of *alpha‐Mann‐2a* at 5 dpi in the midgut and carcass. N = 6‐17. Graphs display the relative expression compared to *RpS6*. Bars represent the median and whiskers depict the 95% confidence intervals. Mann‐Whitney test: * *P* < 0.05.
**Figure S4**. CHIKV infection during RNAi knockdown of *alpha‐Mann‐2a* in the carcass. CHIKV intensity and prevalence were detected by absolute qRT‐PCR in carcass samples at 5‐ and 10 dpi. A. Infection intensity (genome copy number) overtime. Lines mark the median, circles represent CHIKV quantities in individual dsGFP (yellow) or dsAlpha (magenta) tissue samples, and whiskers depict the 95% confidence intervals. Mann‐Whitney test, *P* > 0.07 B. Prevalence (presence or absence) of CHIKV. Bars contain the percentage of mosquitoes uninfected (green) and CHIKV infected (blue). Binary logistical regression for carcass: *P* = 0.48. At 5 dpi N = 7 and at 10 dpi N = 17‐18.
**Table S1**. Statistical analyses for Figures 1 and 6.
**Table S2**. Primers.

## Data Availability

The data that support the findings of this study are openly available in figshare at 10.6084/m9.figshare.13726000.
