# Supplementary material for: Assessing Aedes aegypti candidate genes during viral infection and Wolbachia‐mediated pathogen blocking
Source: Insect Mol Biol. 2022 Feb 14;31(3):356–68. doi: 10.1111/imb.12764 (PMC9081237; doi:10.1111/imb.12764)
Supplement: Supplementary file 1 — Figure S1. Wolbachia mediated DENV‐blocking during alpha‐Mann‐2a RNAi. DENV intensity and prevalence were detected by absolute qRT‐PCR in midgut and carcass samples at 10 and 14 dpi. A. Infection intensity (genome copy number) overtime. Lines mark the median, circles represent DENV quantities in individual dsGFP (yellow) or dsAlpha (magenta) tissue samples, and whiskers depict the 95% confidence intervals. Mann‐Whitney test, not significant. B. Prevalence (presence or absence) of DENV. Bars contain the percentage of mosquitoes uninfected (green) and DENV infected (blue). Binary logistical regression for Midgut: P = 0.09. Binary logistical regression for Carcass: P = 0.07. N = 20‐25. Figure S2. Wolbachia levels 3 days post‐RNAi. The levels of wAlbB in whole body mosquitoes was similar across treatment groups at 3 days post‐dsRNA injection and prior to DENV infection. Mann‐Whitney test, dsAlpha P = 0.7. Bars represent the median and whiskers depict the 95% confidence intervals. N = 3. Figure S3. Expression of alpha‐Mann‐2a following RNAi and infection with CHIKV. A. Levels of alpha‐Mann‐2a expression in mosquitoes at 3 days post‐RNAi (pre‐exposure) compared to control mosquitoes (dsGFP injected). Circles represent individual whole‐body samples. N = 4‐5. B‐C. Expression of alpha‐Mann‐2a at 5 dpi in the midgut and carcass. N = 6‐17. Graphs display the relative expression compared to RpS6. Bars represent the median and whiskers depict the 95% confidence intervals. Mann‐Whitney test: * P < 0.05. Figure S4. CHIKV infection during RNAi knockdown of alpha‐Mann‐2a in the carcass. CHIKV intensity and prevalence were detected by absolute qRT‐PCR in carcass samples at 5‐ and 10 dpi. A. Infection intensity (genome copy number) overtime. Lines mark the median, circles represent CHIKV quantities in individual dsGFP (yellow) or dsAlpha (magenta) tissue samples, and whiskers depict the 95% confidence intervals. Mann‐Whitney test, P > 0.07 B. Prevalence (presence or absence) of CHIKV. [file IMB-31-356-s001.docx]

# Supporting Information
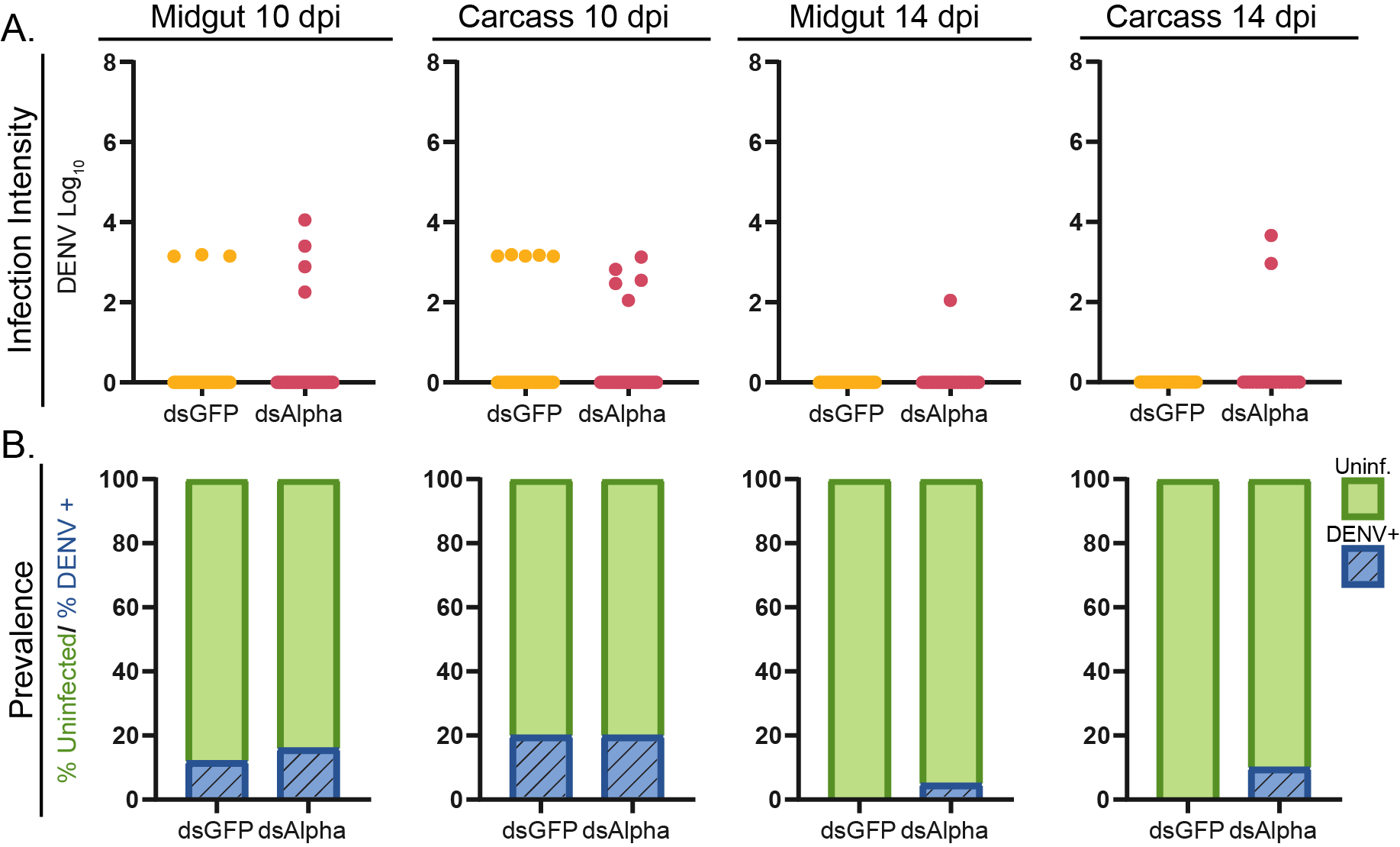


**Supp. Fig. 1: *Wolbachia* mediated DENV-blocking during *alpha-Mann-2a* RNAi****.** DENV intensity and prevalence were detected by absolute qRT-PCR in midgut and carcass samples at 10 and 14 dpi. **A.** Infection intensity (genome copy number) overtime. Lines mark the median, circles represent DENV quantities in individual dsGFP (yellow) or dsAlpha (magenta) tissue samples, and whiskers depict the 95% confidence intervals. Mann-Whitney test, not significant. **B.** Prevalence (presence or absence) of DENV. Bars contain the percentage of mosquitoes uninfected (green) and DENV infected (blue). Binary logistical regression for Midgut: *P* = 0.09. Binary logistical regression for Carcass: *P* = 0.07. N = 20-25.

**
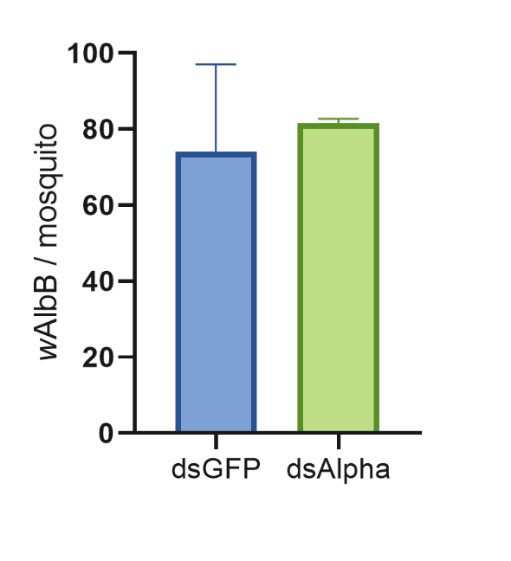
**

**Supp. Fig. 2: *Wolbachia* levels 3 days post-RNAi.** The levels of *w*AlbB in whole body mosquitoes was similar across treatment groups at 3 days post-dsRNA injection and prior to DENV infection. Mann-Whitney test, dsAlpha *P* = 0.7. Bars represent the median and whiskers depict the 95% confidence intervals. N = 3.


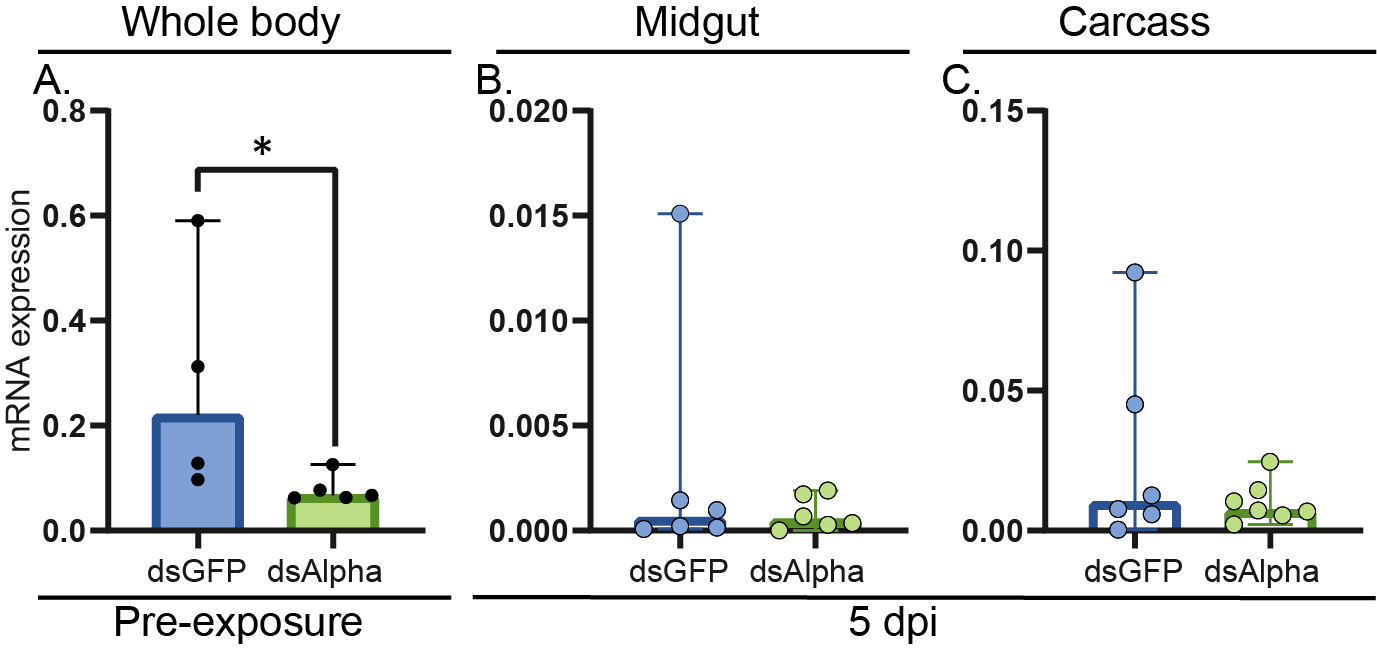


**Supp. Fig. 3: Expression of *alpha-Mann-2a* following RNAi and infection with CHIKV. A.** Levels of *alpha-Mann-2a* expression in mosquitoes at 3 days post-RNAi (pre-exposure) compared to control mosquitoes (dsGFP injected). Circles represent individual whole-body samples. N = 4-5. **B-C.** Expression of *alpha-Mann-2a* at 5 dpi in the midgut and carcass. N = 6-17. Graphs display the relative expression compared to RpS6. Bars represent the median and whiskers depict the 95% confidence intervals. Mann-Whitney test: * *P* < 0.05.


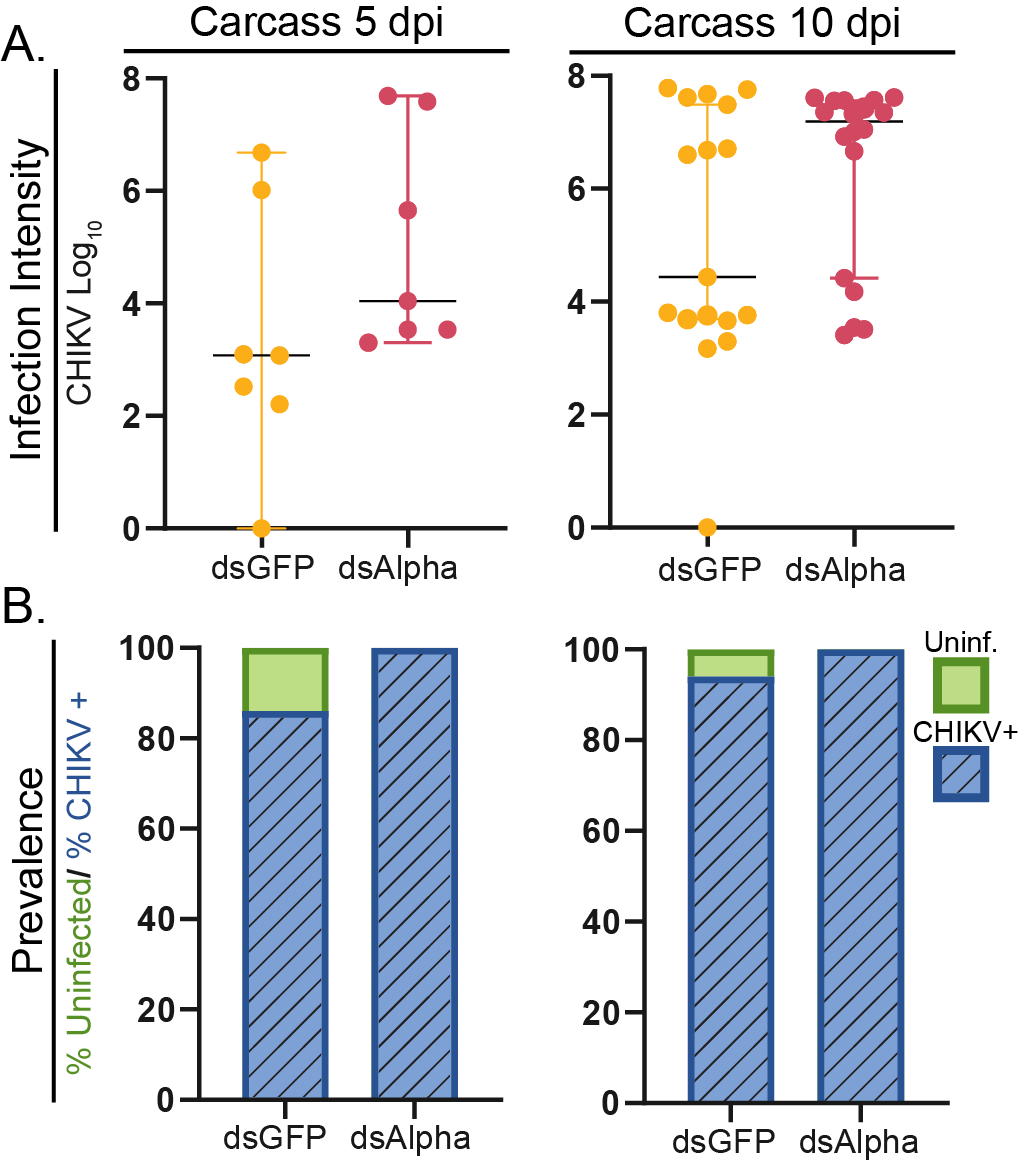


**Supp. Fig. 4: CHIKV infection during RNAi knockdown of *alpha-Mann-2a* in the carcass.** CHIKV intensity and prevalence were detected by absolute qRT-PCR in carcass samples at 5- and 10 dpi. **A.** Infection intensity (genome copy number) overtime. Lines mark the median, circles represent CHIKV quantities in individual dsGFP (yellow) or dsAlpha (magenta) tissue samples, and whiskers depict the 95% confidence intervals. Mann-Whitney test, *P* > 0.07 **B.** Prevalence (presence or absence) of CHIKV. Bars contain the percentage of mosquitoes uninfected (green) and CHIKV infected (blue). Binary logistical regression for carcass: *P* = 0.48. At 5 dpi N = 7 and at 10 dpi N = 17-18.

**Supp. Table 1: Statistical analyses for Figures 1 and 6.**

**CONTINUED: Supp. Table 1: Statistical analyses for Figures 1 and 6.**

**Supp. Table 2: Primers.**

The T7 RNA polymerase promoter sequence tag is lowercase. F = forward, R = reverse. Reverse transcriptase (RT).
